# Radiation of the Tnt1 retrotransposon superfamily in three Solanaceae genera

**DOI:** 10.1186/1471-2148-7-34

**Published:** 2007-03-07

**Authors:** Maria E Manetti, Magdalena Rossi, Ana PP Costa, Andrea M Clausen, Marie-Anne Van Sluys

**Affiliations:** 1 GaTe lab, Departamento de Botânica, Instituto de Biociências – Universidade de São Paulo Departamento de Botânica – IBUSP, Rua do Matão, 277; 05508-090; São Paulo, S.P. Brasil; 2 Faculdade de Ciências Biológicas, Exatas e Experimentais, Universidade Presbiteriana Mackenzie, Brazil; 3 Estación Experimental Agropecuaria Balcarce, Instituto Nacional de Tecnología Agropecuaria (INTA), Argentina

## Abstract

**Background:**

Tnt1 was the first active plant retrotransposon identified in tobacco after nitrate reductase gene disruption. The Tnt1 superfamily comprises elements from *Nicotiana *(Tnt1 and Tto1) and *Lycopersicon *(Retrolyc1 and Tlc1) species. The study presented here was conducted to characterise Tnt1-related sequences in 20 wild species of *Solanum *and five cultivars of *Solanum tuberosum*.

**Results:**

Tnt1-related sequences were amplified from total genomic DNA using a PCR-based approach. Purified fragments were cloned and sequenced, and clustering analysis revealed three groups that differ in their U3 region. Using a network approach with a total of 453 non-redundant sequences isolated from *Solanum *(197), *Nicotiana *(140) and *Lycopersicon *(116) species, it is demonstrated that the Tnt1 superfamily can be treated as a population to resolve previous phylogenetic multifurcations. The resulting RNAseH network revealed that sequences group according to the Solanaceae genus, supporting a strong association with the host genome, whereas tracing the U3 region sequence association characterises the modular evolutionary pattern within the Tnt1 superfamily. Within each genus, and irrespective of species, nearly 20% of Tnt1 sequences analysed are identical, indicative of being part of an active copy. The network approach enabled the identification of putative "master" sequences and provided evidence that within a genus these master sequences are associated with distinct U3 regions.

**Conclusion:**

The results presented here support the hypothesis that the Tnt1 superfamily was present early in the evolution of Solanaceae. The evidence also suggests that the RNAseH region of Tnt1 became fixed at the host genus level whereas, within each genus, propagation was ensured by the diversification of the U3 region. Different selection pressures seemed to have acted on the U3 and RNAseH modules of ancestral Tnt1 elements, probably due to the distinct functions of these regions in the retrotransposon life cycle, resulting in both co evolution and adaptation of the element population with its host.

## Background

Retrotransposons are mobile genetic elements that transpose via an RNA intermediate. They are abundant and widespread components of eukaryotic genomes [1]. There are two major types of retrotransposons: long terminal repeat (LTR) retrotransposons and non-LTR retrotransposons such as LINEs (long interspersed nuclear elements) and SINEs (short interspersed nuclear elements) [2,3]. LTR retrotransposons are abundant in plant genomes and can constitute a very large fraction of the host genome [4,5].

LTR retrotransposons are flanked by LTRs in direct orientation. LTRs are subdivided into U3 (unique 3'RNA), R (repeated RNA) and U5 (unique 5'RNA) regions. Regulatory signals such as promoter, terminator regions and polyadenylation sites are found in the LTRs [2]. Besides the functional importance of these sequences, several studies have reported that the LTRs are the most-rapidly evolving regions of retrotransposons [6,7].

LTR retrotransposons encode a number of proteins derived from the *gag *and *pol *genes, which are usually transcribed as two main mRNAs. The proteins in *pol *are synthesised as a polyprotein that is cleaved by an internal protease activity releasing the internal protein activities endonuclease/integrase, reverse transcriptase and ribonuclease H (RNAseH). The *gag *gene encodes a protein involved in maturation and packaging of the RNA form of the retrotransposon. The life cycle of an element involves transcription by cellular RNA polymerase II, reverse transcription, packaging into virus-like particles, and integration of the cDNA copy back into the genome [2].

The retrotransposon Tnt1, first characterised in tobacco, is one of the few plant retrotransposons for which transpositional activity has been demonstrated [8]. Tnt1-like sequences have been detected in several *Solanaceae *and in *Nicotiana *at least three major groups can be differentiated based on U3 regulatory region sequence divergence giving rise to Tnt1A, B and C subfamilies [9-12]. Retrolyc1 elements detected in tomato share extensive nucleotide similarities to Tnt1 elements except in the U3 region [13,14]. Distinct from Tnt1, Retrolyc1 sequences comprise two subfamilies, Retrolyc1A and B, also distinguished at the regulatory U3 region [14].

The retrotransposon life cycle results in genome amplification and for this reason their activity is tightly controlled by the host. Besides rounds of amplification, host genome size can be scaled down by homologous recombination events within an element or between elements, usually leading to an increase in the copy number of SOLO-LTR sequences [15]. The work presented here aims to address the question of 'how' Tnt1-related sequences differentiated in three Solanaceae genera (*Nicotiana*, *Lycopersicon *and *Solanum*) and if there is any evidence for the occurrence of lateral gene transfer. These questions were addressed in a total of 453 non-redundant sequences using a population model approach based on recent studies of the amplification of *Alu *sequences among human and chimpanzee lineages [16,17].

The results presented here demonstrate the existence of Tnt1 superfamily related lineages in all species studied. Sequences analyses corroborate the hypothesis that the Tnt1-superfamily has evolved through differentiation of the U3 region as previously suggested [12]. RNAseH clustering studies revealed the existence of a major representative RNAseH sequence (α sequence) suggesting the presence of active copies. However, the highly diverse U3 region presents inter- and intra-specific diversification, which supports the hypothesis of a rapidly evolving sequence that can be interpreted as a strategy either to evade negative control by the host or to quickly adapt to the new evolving host genome thus contributing to host fitness.

## Results

### Tnt1-like sequences within different Solanum species

**Figure 1:**
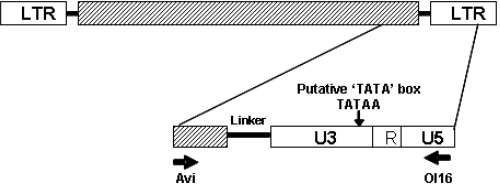
**Schematic representation of the amplified retrotransposon fragment**. LTR: long terminal repeat. RNAseH: ribonuclease H; Linker: noncoding region with PPT (polypurine track); U3: unique 3' RNA region; R: repeat RNA; and U5: unique 5'RNA region. Thick arrows indicate the position of the primers used in the amplifications.

**Figure 2:**
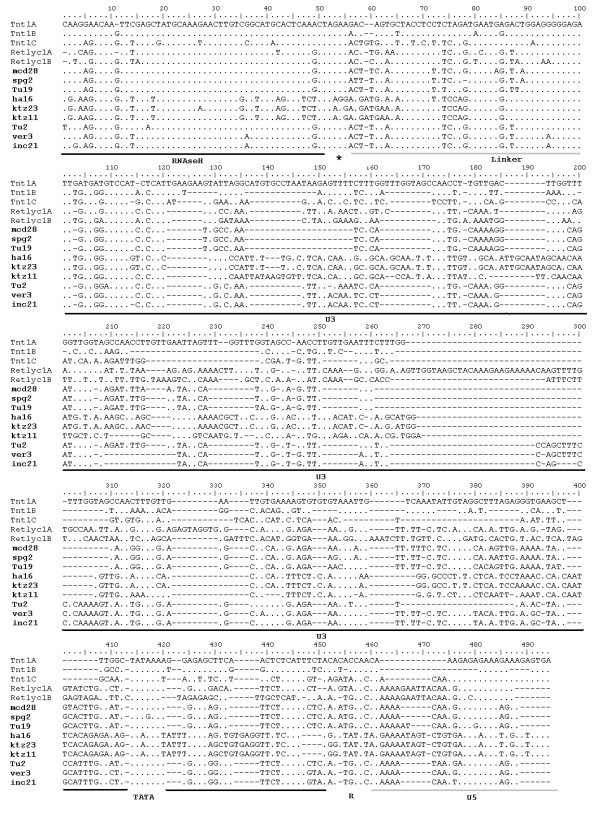
**Sequence alignment of the partial amplified fragments**. Representatives from Tnt1A, B and C; Retrolyc1A and B; and six sequences from Retrosol were included (names in bold). Underlined regions denote: RNAseH (ribonuclease), Linker, U3 (unique 3'RNA region), TATA, R (repeat RNA) and U5 (unique 5' RNA).

The presence of Tnt1-like sequences within 25 wild and cultivated *Solanum *genotypes (Table 1) was assayed by PCR using primers designed on the Tnt1 sequence anchored in the terminal part of the ribonuclease H (RNAseH) domain (Avi), and on the U5 region (Ol16) spanning part of RNAseH, Linker, U3, R and a portion of U5 region (Figure 1). Fragments of the expected size (500 bp) were detected in all genotypes analysed (Table 2). Sequence analysis using the NCBI Blast tool [35] confirmed that all 317 cloned fragments exhibited similarity to the Tnt1 superfamily and were therefore named "Retrosol" to designate them as retrotransposons from *Solanum*. Alignment of nine Retrosol sequences, chosen randomly from seven distinct species, to Tnt1 and Retrolyc1 retrotransposon sequences revealed that the similarity spans the RNAseH, R and U5 regions but not U3 (Figure 2).

**Table 1:** List of *Solanum *species used in this study

accession	section	subsection	Seria	Species	Code	Ploidy
Bal20002	Petota	Estolonifera		*S. brevidens*	Bre	2x
Bal80009		Potatoe	Commersoniana	*S. commersonii *subp.*malmeanum*	Cmm	2x
Bal01280			Yungasensia	*S. chacoense*	Chc	2x
Bal03017				*S. tarijense*	Tar	2x
Bal01113			Megistracroloba	*S. megistacrolobum*	Mga	2x
Bal74326				*S. sanctae-rosae*	Sat	2x
Bal01156			Cuneolata	*S. infundibuliforme*	Ifd	2x
Bal86017			Tuberosa	*S. x doddsii*	Dds	2x
Bal87066				*S. leptophyes*	Lph	2x
Bal90081				*S. microdontum*	Mcd	2x
Bal01121				*S. oplocense*	Opl	4x
Bal86053				*S. x sucrense*	Scr	4x
Bal01007				*S. gourlayi*	Gou	2x
Bal83042				*S. hannemanii*	Ha	2x
Bal73042				*S. incamayoense*	Inc	2x
Bal90044				*S. kurtzianum*	Ktz	2x
Bal74282				*S. okadae*	Oka	2x
Bal03013				*S. spegazzinii*	Spg	2x
Bal74330				*S. vernei*	Vrn	2x
				*S. tuberosum*		
				cultivar *Kennebec*	Ke	4x
				cul.*Huinkul*	Hu	4x
				*S. tuberosum *subp. *andigenum*		
				cul.*Chacarera *109	Cha	4x
				cul *. Moradita *64	Mo	4x
				cul.*Tuni *207	Tu	4x
Bal01109			Acaulia	*S. acaule*	Acl	4x

Accession means collection, year and locality. x in species name, means hybrid.

**Table 2:** Genetic variability parameters of Tnt1-like sequences of *Solanum *wild and cultivated genotypes

**code**	**Domest.**	**total**	**unique**		**size bp**	**π JC**	**I**	**II**	**III**	**premature**	**no stop/cod**
bre	wild	25	13	52%	456(± 7)	0,057	13	-	-	1	5
cmm		3	3	100%	423(± 5)	0,093	1	2	-	-	-
chc		1	1	100%	428	-	1	-	-	-	-
tar		15	9	60%	422(± 14)	0,038	1	8	-	1	-
mga		17	10	58%	439(± 19)	0,171	6	3	1	-	-
sat		7	6	85%	440(± 4)	0,069	4	2	-	-	-
inf		23	10	43%	428(± 9)	0,053	4	6	-	2	-
dds		1	1	100%	435	-	1	-	-	1	-
lph		1	1	100%	436	-	1	-	-	-	-
mcd		2	2	100%	428(± 11)	0,078	1	1	-	-	-
opl		2	2	100%	419(± 2)	-	-	2	-	1	-
scr		12	12	100%	463(± 11)	0,132	6	5	1	1	1
gou		3	3	100%	430(± 8)	0,093	2	1	-	1	-
ha		19	12	63%	428(± 9)	0,249	2	7	3	-	-
inc		21	11	52%	421(± 4)	0,041	2	9	-	3	-
ktz		18	14	77%	443(± 8)	0,287	7	-	7	-	-
oka		16	7	43%	434(± 10)	0,273	2	3	2	-	-
spg		20	9	45%	433(± 7)	0,055	5	4	-	3	-
vrn		12	4	27%	429(± 9)	0,072	2	2	-	-	-
Ke	cult.	8	8	100%	426(± 14)	0,078	2	6	-	-	-
Hu		4	4	100%	421	0,026	-	4	-	-	-
Cha		23	17	74%	424(± 7)	0,064	7	10	-	-	-
Mo		29	17	58%	423(± 6)	0,057	6	11	-	-	-
Tu		17	14	82%	428(± 10)	0,058	8	6	-	-	-
acl	wild	18	7	38%	419(± 19)	0,191	3	3	1	-	4
**total**		317	197	62%			87	95	15	14	10

Note. Code refers to the codes use in table 1 for each species and cultivars. Domestication (Domest.) status: wild or cultivated (cult.) genotypes. Total: indicates total number of sequences obtained; unique genotypes: total number of different sequences and percentage; size: average fragment size in bp (size variation); πJC indicates nucleotide diversity with Jukes and Castor's (1969) correction; I, II and III refers to the sequence identity group obtained from the tree presented in figure 3 and numbers in these columns indicates numbers of sequences; premature: refers to number of sequences with premature stop codon and no stop/cod: refers to number of sequences with no stop codon in the RNAseH domain.

Intra-genotype redundancy was eliminated and only unique sequences within species were considered for further sequence studies (Table 2). As these species do not have well-characterised genomes, it cannot be determined whether the amplified Retrosol fragments represent an unbiased random sample of sequence diversity. However, for a survey of the evolution of this element family, this approach seems appropriate as it was also used previously in the characterisation of the Tnt1 and Retrolyc1 families. A few sequences had a premature stop codon and others no stop codon at all in the RNAseH domain (Table 2).

The nucleotide divergence index (πJC), with Jukes and Castor's correction, was calculated for the full-length fragment sequence within each genotype. Numbers range from 0.026 to 0.287, and allow the genotypes to be classified within three sets (Table 2). The most diverse displays πJC values ≥ 0.2 and encompassed *S. hannemanii, S. kurtzianum *and *S. okadae *species. The second, involving *S. acaule, S. megistacrolubom *and *S. xsucrense*, presented values between 0.1 and 0.2; while the third, involving all remaining species had an πJC index of < 0.1. It is worth pointing out that cultivated potatoes (*S*.*tuberosum *cultivar Huinkul, Kennebec, and subsp. *andigenum *cul. Moradita, Chacarera and Tuni) were in the least diverse group.

### Phylogenetic Relationships of Solanaceae Tnt1-related sequences

**Figure 3:**
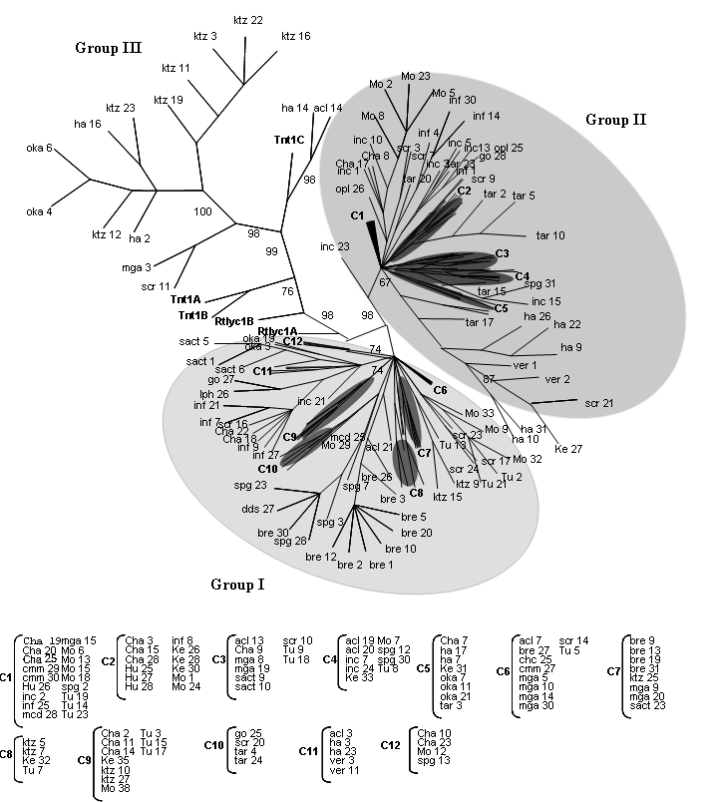
**Phylogenetic analysis of Tnt1 Solanaceae superfamily**. Phylogenetic analysis was performed with 197 sequences amplified from different species of *Solanum *(species are indicated by a code cited in Table 1 and different clones are indicated by numbers), and representative sequences from Tnt1 A, B and C, and Retrolyc1 A and B. The aligned nucleotide sequences span the last fragment of the RNAseH domain, the linker, U3, R and part of the U5 region.

To visualise the relationship between all Solanaceae Tnt1-related sequences, a phylogenetic analysis was performed using all full-length fragments amplified. Retrosol sequences were aligned, together with representatives of Tnt1A B and C, and Retrolyc1A and B subfamily sequences. Three groups were obtained supported by a bootstrap of 98% or higher (Figure 3). Group I contains 87 sequences, including both wild and cultivated species and all 15 sequences amplified from *S. brevidens *(Table 2). Nucleotide identity between sequences varies from 80–90%. Group II has 95 sequences from wild and cultivated species (Table 2) and nucleotide identity is higher than 90% between sequences. Group III has 15 sequences belonging to 6 wild species: *S. acaule*, *S. hannemanii*, *S. kurtzianum*, *S. okadae *and *S. xsucrense *(Table 2) with variable nucleotide identity ranging from 60–95%. The Tnt1 C sequence clusters to Group III while Tnt1 A, B and Retrolyc1 A and B form a bridge between Groups I and II and the other sequences.

**Figure 4:**
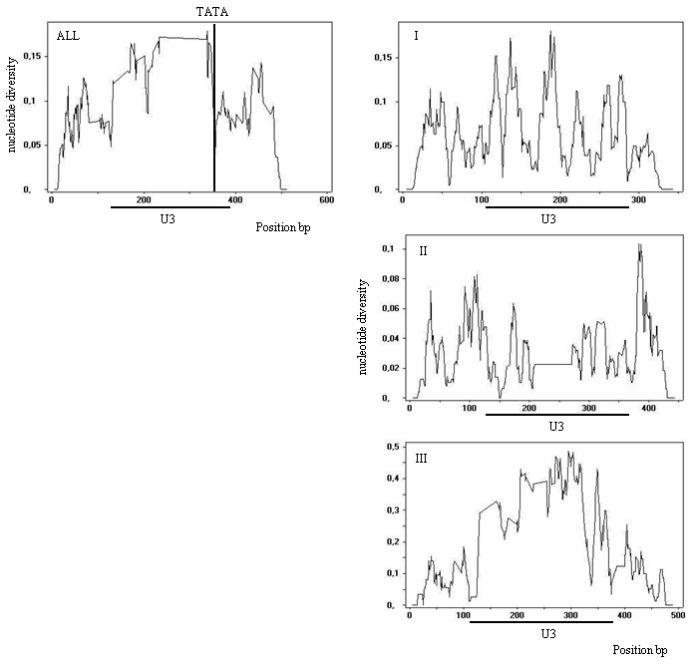
**Sequence divergence distribution along the amplified fragments**. The values on the x-axis correspond to the nucleotide position of the fragment amplified. The values on the y-axis are the nucleotide diversity (π) measure, the percentage of divergent nucleotides relative to the number of informative bases, calculated using a sliding window of 10 bp and a step of 1 bp, by the DnaSp program [31]. The position of the TATA box and U3 are indicated. Data are shown for all sequences amplified and for pair-wise comparisons within groups without gaps.

The πJC index calculated for all unique Retrosol sequences was 0.087 ± 0.0000571. Considering Retrosol phylogenetic groups separately, the nucleotide variability is lower for sequences of Group II (0.030 ± 0.0000016), and for Group I (0.063 ± 0.0000035). Group III has the highest value (0.220 ± 0.0009489), probably representing more than one group of related sequences. Nucleotide diversity is not uniformly distributed amongst the amplified fragments. When a comparative sliding window for nucleotide diversity is applied on all aligned sequences or within each group, as shown in Figure 4, higher substitution rates are seen to be in the U3 region. However, when nucleotide diversity distribution was analysed within each group, Group II showed a less diverse U3 region compared to the other groups, while Group III sequences exhibited the highest diversity in the U3 region. The data support the hypothesis that *Solanum *species harbour at least three versions of U3 regulatory regions, although based on previous Tnt1 superfamily U3 divergence studies [12], Group III could represent more than one version.

### NETWORK analysis of Tnt1 superfamily ribonuclease H

Network approaches have been designed to investigate relationships between closely related sequences, allowing identification of persistent ancestral nodes, multifurcations and reticulations, which are not resolved when applying a conventional phylogenetic package [19]. In the network approach, the most-represented identical sequences – named α or master copy – occupy a central position in the net with high numbers of branches suggesting that the sequence represents part of an active element. This approach is based on the parsimony principle, which connects data sets with the minimum number of evolutionary steps. Reticulations on the net could result from recombination and/or homoplasy [18,19].

**Figure 5:**
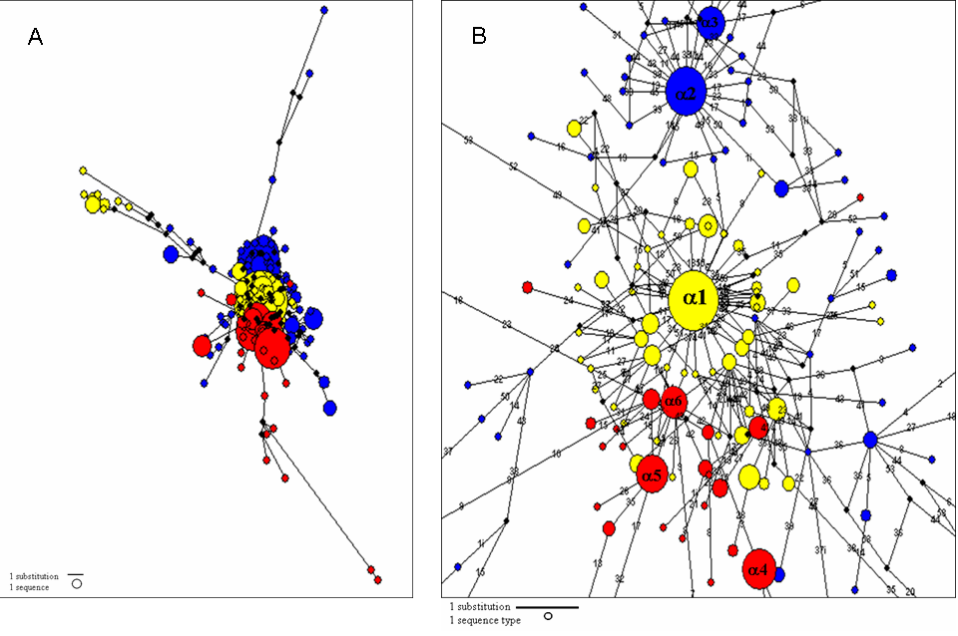
**Median-joining networks of the RNAseH coding region**. The network was constructed using 443 Tnt1 superfamily sequences. Circles denote sequence types: yellow circles denote sequences types from Retrosol, blue circles denote sequences from Tnt1, and red circles denote sequences from Retrolyc1. (A) The size of the circle is proportional to the number of sequences, with one sequence type indicated in the bottom-left corner. Lines denote substitutions; with one-step distance indicated in the bottom-left corner. Reconstructed nodes are identified as black circles; they are absent in the dataset because they were not sampled or lost. (B) Zoom of the median-joining networks of the RNAseH coding region. The size of the circles is proportional to the number of sequences, with one sequence type indicated in the bottom-left corner. Lines denote substitutions and numbers on the lines indicate the substitution position on the sequence. One-step distance is indicated in the bottom-left corner. The labels in the nodes illustrate the nomenclature used to refer to the original master sequence of each genus, called α type: α1 is the master copy from Retrosol, α2 and α3 are from Tnt1 and α4, α5 and α6 are from Retrolyc1.

Considering the Tnt1 superfamily of retrotransposons in the genera *Solanum, Lycopersicon *and *Nicotiana *as a population of sequences with a common ancestor, a Network study was carried out with the aim of testing the hypothesis that a common α master sequence exists in Tnt1-superfamily. The 53 nucleotides corresponding to the last portion of the RNAseH coding domain from a total of 453 Tnt1 like-sequences (197 Retrosol sequences reported in this study, 140 Tnt1 sequences characterised from 7 *Nicotiana *species and 116 Retrolyc1 sequences from 4 species of *Lycopersicon*) were used for network analysis. The resulting network (Figure 5a) revealed that Tnt1, Retrolyc1 and Retrosol did not share any RNAseH sequence as revealed by the absence of shared nodes, although different species within each genus did share a master sequence.

In Retrosol sequences, the unique α node observed (α1) represents 25.5% of all Retrosol sequences and encompasses *S. hannemanii, S. incamayoense, S. infundibuliforme, S. microdontum, S. megistacrolobum, S. okadae, S. sanctarosae, S. spegazzinii, S. tarijense, S. x sucrense, S. vernei *and all *S. tuberosum *cultivars. It is worth mentioning that 46 of the 50 α1 sequences belong to Group II, which is the least diverse phylogenetic group. Moreover, within α1 group it was possible to identify identical U3 regions belonging to different species and cultivars. A total of 13 non-redundant *S. brevidens *sequences are connected with the α sequence in the net through a nucleotide change. None of the sequences belonging to group III appear in the α1 cluster; group III sequences are located at a more distant position in the net, suggesting a more distant relationship with the core sequence and probably do not originated from the α1 node.

Tnt1 sequences originated from two α nodes: α2 with 24% (34 sequences) and α3 with 12 % (17 sequences) of Tnt1 sequences (Figure 5b). α2 sequences belonged to: *Nicotiana tabacum*, *N. debneyi*, *N. benthamiana, N. plumbaginifolia *and *N. sylvestris*, embracing the three Tnt1 sub-families (A, B and C). α3 clustered sequences from *Nicotiana tabacum, N. glauca, N. debneyi*, *N. benthamiana, N. plumbaginifolia *and *N. sylvestris*, grouping Tnt1 subfamilies A and C. It is interesting to note that Tnt1-94, for which transpositional activity has been demonstrated [8], grouped in the α3 node.

Retrolyc1 showed three different α nodes: α4 with 18.9% (22 sequences), α5 with 17% (20 sequences) and α6 with 13% (15 sequences) of Retrolyc1 sequences (Figure 5b). α4 include sequences from *Lycopersicon pimpinellifolium *and *L. esculentum *– all from the Retrolyc1 B subfamily. α5 has sequences that belong to *L. peruvianum *and to the Retrolyc1 A subfamily, while α6 has 14 sequences that belonged to *L. peruvianum *and only one from *L. hirsutum *– all from Retrolyc1 A. Identical U3 sequences were shared by different species in the α4 node.

Other data nodes with different numbers of sequences are connected with the central α1, α2, α3, α4, α5 and α6 nodes, suggesting that these members could be active and could contribute to the expansion of each element inside its corresponding genus (Figure 5b). The network did not show any excess of multidimensional reticulations so the influence of homoplasy can be considered as negligible [19].

## Discussion

Genome expansion and contraction resulting from rounds of amplification/deletion of transposable elements is becoming accepted as a major component influencing the diversification of eukaryote genomes, as suggested by studies in particular plant and animal species [15,19]. Genome sequencing project also shed light on the ancient associations of known transposable element families with plant genomes. For example, in the work described by Rossi *et al*. (2004) [20], a phylogenetic study on Mutator-like elements (MULE) identified in sugarcane revealed the existence of 4 MULE lineages in Angiosperms prior to the divergence of Monocots and Eudicots.

The retrotransposon Tnt1 was initially characterised in *Nicotiana tabacum *and then detected by probe hybridisation in other Solanaceae genomes [8]. Ten years later, a new Tnt1-like element, Retrolyc1, was characterised in *Lycopersicon peruvianum *[13]. In this study we report a new family member, Retrosol, present in several wild and cultivated *Solanum *genotypes from South America. These results suggest an early association of the Tnt1 superfamily in the evolution of this plant family (Solanaceae).

In addition to its functional importance, the LTR is one of the most rapidly evolving retrotransposon regions. LTRs contain important functional regions such as terminal segments, promoter and enhancer elements, RNA processing signals, and it is recognised for the integration process. In the *BARE*-1 retrotransposon of barley, the whole LTR is heterogeneous [7], while in Tnt1 superfamily the variability is targeted specifically to the U3 region of the LTR [12]. The three subfamilies of Tnt1 retrotransposons, as well as Retrolyc1 subfamilies, are also differentiated mainly by the U3 region. Retrosol sequences present the same structure, showing an accumulation of INDELs and SNPs targeted specifically to the U3. Thus, the hyper variability of the U3 region in the Tnt1-superfamily seems to be a general phenomenon of this element's evolution within Solanaceae species.

Three clades emerged when applying phylogenetic methods to analyse amplified Retrosol sequences from *Solanum *sequences. Groups I and II are consistent because they represent cohesive versions of U3. In contrast, Group III encompasses the most diverse sequences, probably due more than one U3 version being represented. No correlation is observed between a particular U3 version and genotype, wild or cultivated species, or geographical distribution (Tables 1, 2). These results suggest that the amplification and differentiation of Retrosol occurred prior to *Solanum *speciation.

A population model was applied in this work to evaluate the relationships among Tnt1-derived elements in *Solanum, Lycopersicon *and *Nicotiana *with the aim of testing the existence of a unique master copy. To analyse the Network results, the three models of expansion proposed by Cordaux *et al *(2004) [19] for *Alu *subfamily in humans and primates were considered: the single 'master gene' model, the intermediate model and the transposon model. According to the 'master gene' model, a single α-type element generates all other subfamily members, leading to a star-like relationship with all inactive copies derived from the α element. The contrasting transposon model postulates that all element members can be active in producing new copies, resulting in the lack of a radiation structure from the α central node. The intermediate model suggests that several members are active and contribute to expansion. Relationships in this latter model are expected to be partly star-like, but also with different proportions of elements that are not connected directly with the central element depicted as the "α "-sequence.

The RNAseH network supports different models of expansion depending on the element. In addition, RNAseH sequence types, represented by nodes in the net, are distinctive for each genus. Retrosol and Tnt1 show a star-like topology, with the central α node corresponding to the most frequent sequence type, although Tnt1 presented more than one α node. Other nodes not directly connected with the central α node are also found, indicating that the sequence type immediately downstream is also capable of amplification. The unique Retrosol α node is represented in 16 out of the 25 genotypes analysed, taking into account that for most of the other 9 genotypes only a few sequences were cloned. In summary, the model of expansion of Tnt1 in *Nicotiana *sp. and Retrosol in *Solanum *sp. best suits the intermediate model. In contrast, Retrolyc1 present a model with the absence of a radiating structure from a central node and with more than two α nodes, suggesting that many family members are capable of producing new ones. However, these results cannot be used to make conclusions regarding Retrolyc1 evolution as the sequences used in this study were obtained from only a few species of *Lycopersicon*.

The α1 in Retrosol encompasses 92% of Group II sequences, representing 50% of the total Group II sample. As mentioned above, Group II presents the least variable U3 region (Figure 4), and several Group II sequences amplified from different genotypes have identical U3 regions. Thus, sequences with identical RNAseH and U3 regions transposed prior to the divergence of the host species. This activity is recent enough that element copies should not have accumulated mutations or deletions in the U3 variable region. All these lines of evidence point out the protagonist role of Group II in Retrosol expansion as the transpositionally active lineage.

RNAseH is conserved in Retrosol elements across host species from the same genus, while the U3 region has evolved rapidly, in agreement with the concept of modular evolution. The conservation of the RNAseH module at the genus level is related to its biological function, in which the enzyme has to interact with other molecules. RNAseH is involved in degradation of the original RNA template, generation of a polypurine track, and final removal of RNA primers from the newly synthesised minus and plus DNA strands [21]. These functions are necessary for the transpositional activity of the element. On the other hand, the U3 module evolved rapidly in accordance with its promoter function. New promoter sequences may permit the expression of the element in diverse environmental conditions, increasing the survival potential of the element in the host and/or the probability of overcoming host transcriptional silencing efforts. In addition, the host may gain an advantage in generating genome diversity by insertion of new retrotransposon copies, increasing its own environmental fitness. Co-evolution and co-adaptation of retrotransposon with its host genome is expected to play a particularly important role in the long-term survival of these genetic elements [22].

The Solanaceae is one of the largest flowering plant families, embracing 96 genera. It has a world-wide distribution but the greatest concentration of genera and species is found in South and Central America. About three-quarters of these genera and around half of the total number of species are found there; strongly suggesting that the family itself originated in the part of the ancient land mass that later became South America [23]. In order to analyse the evolution of the Tnt1 superfamily, species from three genera were considered: *Solanum*, *Lycopersicon *and *Nicotiana*. *Solanum *is one of the largest genera in Angiosperms and accounts for all wild and cultivated tuber-bearing species originating in the high Andes of southern Peru and northern Bolivia. *Lycopersicon *includes the tomato and its wild relatives, which are confined mainly to Chile, Peru and Ecuador. *Nicotiana *encompasses cultivated tobacco and wild species found mainly in South America and Australia. These three genera originated in South America but the present species distribution reflects several dispersal phenomena [24]. Could Tnt1-related elements be a determinant in the speciation of the Solanaceae?

As proposed by Grandbastien et al [12], the composition of present element populations results from the adaptive response of the ancestral Tnt1 population to different hosts. This adaptive element response refers to appropriate expression patterns, efficient mechanisms of replication and integration, and insertion into non-deleterious genomic sites. Ancestral copies exhibiting such adaptive responses were selectively amplified during or after the radiation of Solanaceae. On the other hand, non-transcribed copies would have been rapidly inactivated and lost. In Retrosol elements, no correlation is found between host species, U3 region or RNAseH sequence type, suggesting that diversification of *Solanum *species was not a determinant of this retrotransposon population. Similar results have been reported for Tnt1 [10] and Retrolyc1 [14].

## Conclusion

The use of a population model based on the network approach to evaluate the nature of the association of Tnt1-related sequences in Solanaceae supports the existence of an ancestral element rather than the occurrence of recent lateral gene transfer. The modular nature of such retrotransposons is clearly demonstrated by the finding that protein functions, represented here by RNAseH, are characterised as slowly evolving sequences, while the U3 regulatory region is under a less restrictive evolutionary force. Current populations of Tnt1, Retrolyc1 and Retrosol most likely result from their vertical transmission in Solanaceae. The molecular basis that drives U3 differentiation, and whether the rapidly evolving sequence is the result of a strategy to evade host negative control or an attempt to quickly adapt to the new evolving genome and contribute to host fitness, remains to be demonstrated.

## Methods

### Plant Material and DNA extraction

*Solanum *seeds and mini-tubercles were kindly provided by the Germplasm collection of INTA-Balcarce (Argentina). Details of species and cultivars collected from locations in Argentina and Bolivia are presented in Table 1. These localities are included in the geographical distribution of the species. *Solanum brevidens *was included in the study as the only species not producing tubers.

Fresh leaf material of commercial cultivars Kennebec and Huinkul were obtained from *in vitro *clones (INTA-Castelar-Argentina), while local Argentinian cultivars Chacarera 109, Moradita 64 and Tuni 207 were obtained from minitubers. The other *Solanum *wild species were germinated from seeds. All material was maintained and processed at Departamento de Botânica-IBUSP (Brazil).

Seeds were surface-sterilised with 10% (v/v) commercial bleach for 15 min, followed by three rinses with sterile water. Treatment with giberellic acid was performed for 24 hrs under dark conditions to break dormancy. Plants were grown in vitro in Murashige and Skoog (MS) basal medium supplemented with 20% sucrose under an 18 h photoperiod, and 22°C day/18°C night temperature regime, in a Percival growth chamber. After three months, leaves were harvested for DNA extraction. Minitubers were grown in soil under the same conditions. Total DNA was extracted as previously described [25] with an average yield of 600ng/mg of fresh tissue.

### PCR amplification, cloning and sequencing

PCR amplifications were performed under standardised conditions using 300 ng of genomic DNA in a final volume of 100 μL. The reaction mix contained 0.23 μM of each primer (Avi; 5'GCTGACCAAGGTGGTAC3'; Ol16; 5'TTCCCACCTCACTACAATATCGC3'), 10 mM dNTPs, 1.5 mM MgCl2, and 5 U of *Taq *DNA polymerase (Invitrogen Brazil). Samples were amplified for 35 cycles with the following cycling conditions: denaturing at 94°C for 45s, annealing at 50°C for 45s, and extension at 72°C for 1 min. An initial denaturing step at 94°C for 5 min and a final extension step for 10 min were also performed.

PCR-amplified fragments were resolved by electrophoresis on agarose gels. Following purification (QIAquick Gel Extraction Kit-QIAGEN), fragments were cloned into the vector pGEM-T-easy (Promega) following the manufacturer's recommendations.

A total of 317 clones were sequenced in both directions using Big Dye Terminator Kit (Applied Biosystems) on an ABI 3700 sequencer (Applied Biosystems). All consensus sequences were generated with a quality Phred ≥ 20. Clone sequences were assembled with the Phred/Phrap/Consed package with a final error rate of < 1 bp/10 kb [26-28].

### Sequence alignment and analysis

The identity of the sequences obtained was confirmed by a BLAST search [29] [available at the National Center for Biotechnology Information (NCBI) Bethesda, Md.]. Consensus sequences were aligned using the CLUSTAL W multiple-alignment program (version 1.5) [30] and manual adjustments of the alignments were performed when necessary.

Sequence divergence was analysed using the DnaSp program [31]. The PAUP program [32] was used to perform phylogenetic analysis based on the distance method with a nucleotide Kimura 2-parameters model. Support for groups was evaluated with 1000 bootstrap replicates. Nucleotide identity between all pairwise sequences was calculated with Strecher from EMBOSS [33].

For Network clustering, sequences were aligned using DNA Alignment 1.1.21 software and relationships were analysed using NETWORK 4.1 [34] both software available at fluxus-engineering.com [36].

The Tnt1 sequences were deposited with the GenBank nucleotide sequence database under the following accession numbers: [GenBank: AJ227998–AJ228017] for *Nicotiana tabacum *tab1-tab20, [GenBank: AJ228018–AJ228037] for *N. sylvestris *syl1-syl20, [GenBank: AJ228038–AJ228057] for *N. plumbaginifolia *plumb1-plumb20, [GenBank: AJ228058–AJ228077] for *N. benthamiana *ben1-ben20, [GenBank: AJ228078–AJ228097] for *N. debneyi *deb1-deb20, [GenBank: AJ228098–AJ228117] for *N. glauca *glau1-glau20, [GenBank: AJ228118–AJ228137] for *N. tomentosiformis *tom1-tom20, and for Tnt1-94, [GenBank: X13777]. Retrolyc1 sequences from *Lycopersicon peruvianum*, *L. hirsutum*, *L. pimpinellifolium *and *L. esculentum *were obtained from Araujo *et al*. 2001 [14], and from this study.

## Authors' contributions

MEM carried out the molecular genetic studies, analysed and interpreted the data and drafted the manuscript. MR analysed and interpreted the data and drafted the manuscript. APPC and AMC revised the manuscript. MAVS conceived the study, participated in its design and coordination, analysed and interpreted the data, and contributed to writing the manuscript. All authors read and approved the final manuscript.
